# Early Blood Perfusion Surgical Strategy for Type A Acute Aortic Dissection With Left Coronary Malperfusion

**DOI:** 10.7759/cureus.90320

**Published:** 2025-08-17

**Authors:** Yoshun Sai, Keita Kikuchi, Joji Ito

**Affiliations:** 1 Department of Cardiovascular Surgery, Tokyo Bay Urayasu Ichikawa Medical Center, Urayasu, JPN

**Keywords:** aortic dissection complications, aortic dissection surgery, coronary artery bypass grafting (cabg), malperfusion, type a acute aortic dissection

## Abstract

Acute type A aortic dissection (AAD) with left coronary artery malperfusion is a critical condition that requires prompt reperfusion to prevent myocardial damage. We report the case of a 66-year-old man with AAD and left coronary malperfusion who underwent emergency surgery. A saphenous vein graft was anastomosed to the LAD before aortic cross-clamping, enabling early reperfusion from the cardiopulmonary bypass circuit. Myocardial function improved markedly during surgery. The patient was successfully weaned from cardiopulmonary bypass and discharged on postoperative day 20 with improved cardiac function. This case highlights the benefit of initiating coronary perfusion prior to aortic cross-clamping as a strategy to improve outcomes in AAD with coronary malperfusion.

## Introduction

Acute type A aortic dissection (AAD) is a life-threatening cardiovascular emergency that demands immediate medical intervention, as the mortality rate increases dramatically with each hour of delay [1]. If untreated, AAD can lead to a mortality rate as high as 50% within the first 48 hours [1]. Coronary malperfusion, defined as compromised blood flow through one or more coronary arteries due to the dissecting process, occurs in approximately 10% to 15% of patients with AAD and is strongly associated with adverse outcomes [2]. In particular, involvement of the left coronary artery can precipitate extensive myocardial ischemia and infarction, potentially resulting in hemodynamic collapse or fatal arrhythmias [3].

Early recognition of coronary malperfusion is therefore crucial, as prompt intervention aimed at restoring coronary blood flow significantly improves survival and decreases the risk of irreversible myocardial damage [3]. Surgical repair is typically the definitive treatment; however, depending on a patient’s hemodynamic stability and the location or extent of the dissection, additional procedures such as coronary artery bypass grafting or percutaneous coronary stent placement may be necessary to ensure adequate reperfusion [4]. In our institutional strategy for AAD with left coronary malperfusion, we prioritize establishing cardiopulmonary bypass and performing on-pump beating coronary artery bypass grafting (CABG) prior to aortic cross-clamping in order to expedite targeted coronary reperfusion while avoiding additional global myocardial ischemia.

Furthermore, contemporary registry and population-based data underscore the time-sensitive lethality of AAD: in the International Registry of Acute Aortic Dissection (IRAD) analysis by Harris et al. (2022), 48-hour mortality was 23.7% for medically managed patients (≈0.5%/h) and 4.4% in the surgical cohort following hospital arrival, highlighting the critical impact of timely surgery [5]. Complementary all-comers data for non-surgically treated AAD demonstrate very high early mortality (e.g., 47.3% at 24 h and 55.0% at 48 h), reinforcing the imperative for rapid diagnosis and reperfusion-oriented strategies when coronary malperfusion is present [6].

This case report describes the successful management of a patient with AAD complicated by left coronary malperfusion, emphasizing the importance of prompt diagnosis, early reperfusion, and definitive surgical repair in optimizing patient outcomes and minimizing myocardial injury in this critical scenario.

## Case presentation

**Figure 1:**
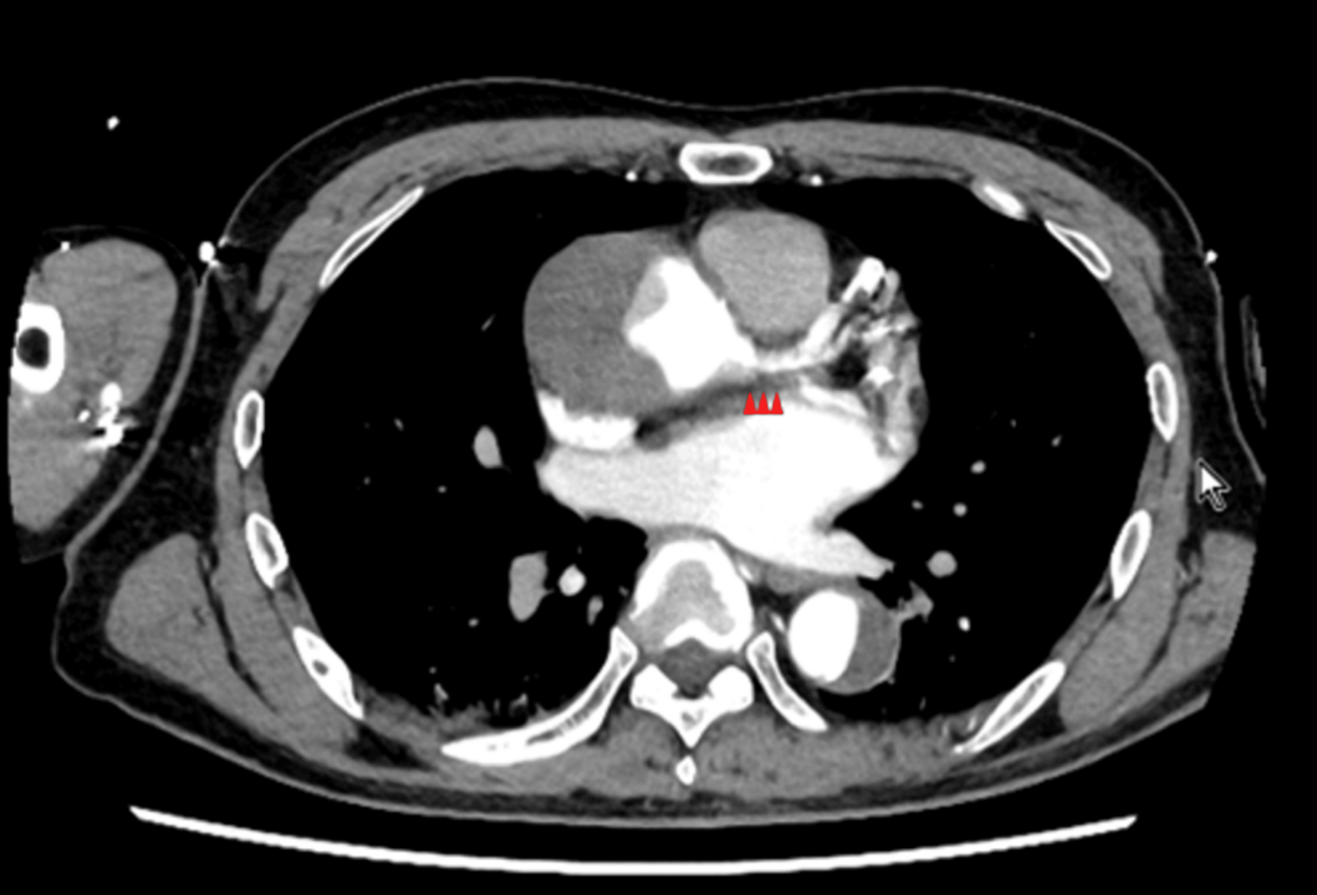
Contrast-enhanced CT demonstrating the presence of thrombotic occlusion in acute type A aortic dissection. The false lumen compresses the ostium of the left main trunk. Arrowheads indicate the false lumen exerting pressure on the ostium of the LMT. CT, computed tomography; LMT, left main trunk.

A 66-year-old man experienced a sudden loss of consciousness while playing golf. He was immediately transported to a local hospital, where emergency contrast-enhanced computed tomography revealed an acute AAD (Figure 1). The patient was referred to our hospital for further management following endotracheal intubation. He arrived at our hospital approximately 60 minutes after the onset of symptoms on the golf course.

**Figure 2:**
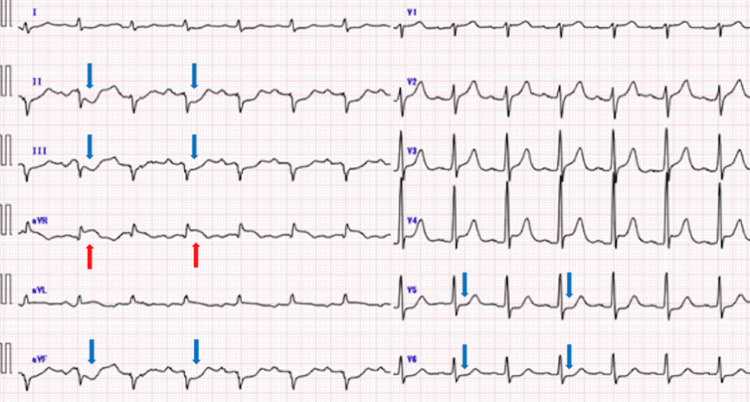
Initial 12-lead electrocardiogram showing ST-segment elevation in lead aVR (red arrows) and ST-segment depression in leads II, III, aVF, and V5–6 (blue arrows).

On arrival at our institution, the patient presented with the following vital signs: Glasgow Coma Scale score E1VTM1, blood pressure, 55/35 mmHg; heart rate, 82 beats per minute; and oxygen saturation (SpO_2_), 95%. An initial 12-lead electrocardiogram showed ST-segment elevation in lead aVR and ST depression in leads II, III, aVF, and V5-6 (Figure 2), suggesting myocardial ischemia.

Transesophageal echocardiography (TEE) revealed diffuse hypokinesis with an estimated ejection fraction (EF) of 20% (Video 1) and mild aortic regurgitation. These findings were consistent with severe left ventricular dysfunction secondary to acute AAD with left coronary malperfusion.

**Video 1:** Preoperative transesophageal echocardiography (TEE) of type A acute aortic dissection with left coronary malperfusion.

**Figure 3:**
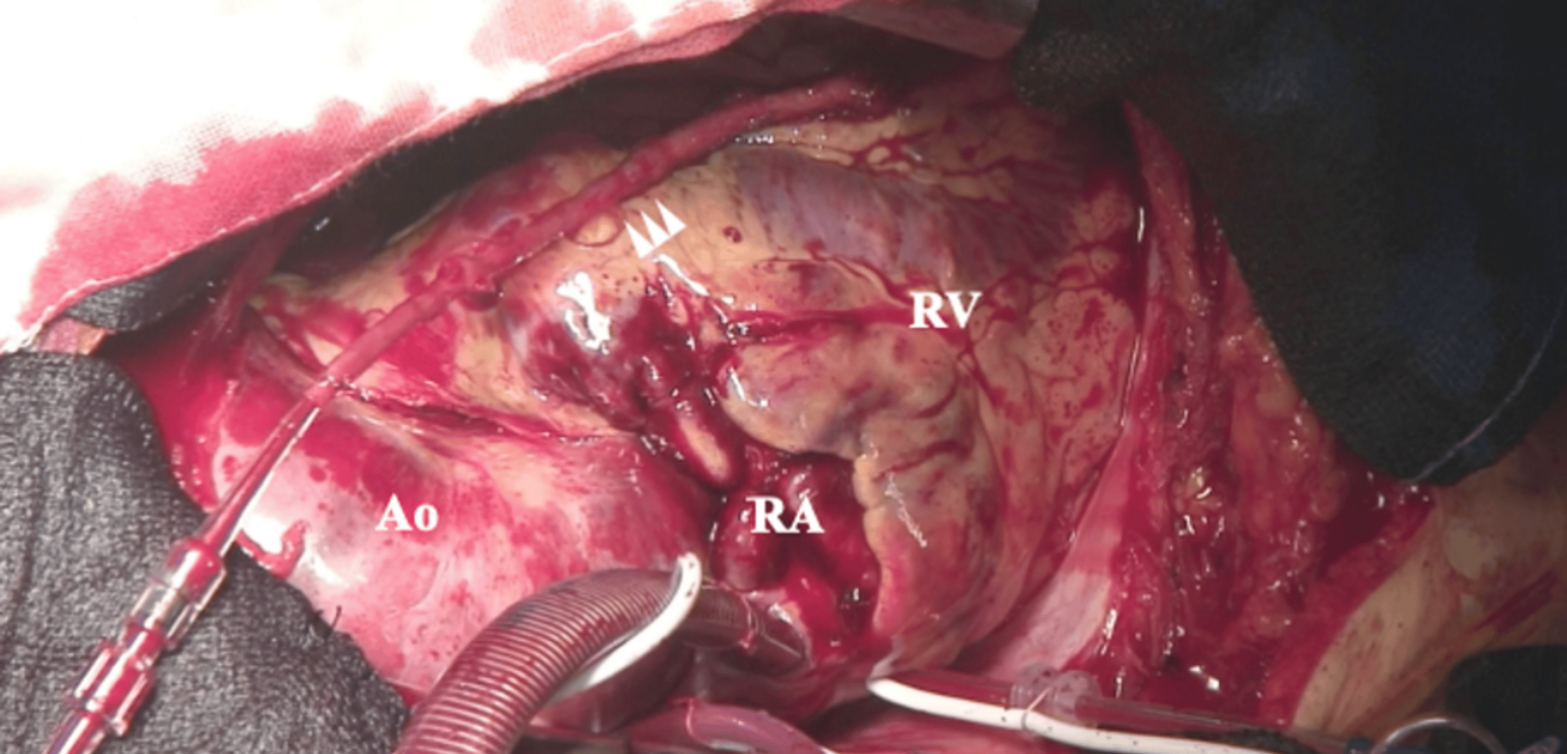
Intraoperative view of the saphenous vein graft anastomosis to the left anterior descending artery. Anastomosis of the saphenous vein graft to the left anterior descending artery (LAD) to initiate cardiopulmonary bypass (CPB) perfusion before cross-clamping. Ao, aorta; RA, right atrium; RV, right ventricle; arrowheads, saphenous vein graft anastomosed to the LAD artery.

The patient was immediately transferred to the operating room for an emergency surgery. The procedure included ascending aortic replacement and coronary artery bypass grafting using a saphenous vein graft to the left anterior descending artery (LAD). Cardiopulmonary bypass (CPB) was performed via the right femoral artery and right atrial cannulation. A vent tube was inserted into the left ventricle through the right superior pulmonary vein, and a retrograde cardioplegia cannula was placed in the coronary sinus. The primary objective was to relieve myocardial ischemia caused by the compression of the left main trunk (LMT) by the false lumen. A saphenous vein graft was harvested from the left thigh and anastomosed to the LAD artery to enable blood perfusion from the CPB to the LAD artery (Figure 3). The harvesting of the great saphenous vein required approximately 10 minutes, and the anastomosis itself required another 20 minutes. Following reperfusion, myocardial stiffness markedly improved. Hence, it took about 30 minutes from the start of surgery to complete the bypass and achieve reperfusion, which occurred 63 minutes after the patient’s arrival at our hospital.

After cross‐clamping the aorta, the adventitia was incised, and thrombus within the false lumen was meticulously debrided. The aorta was then trimmed just above the sinotubular junction, and an 8‐mm felt strip was placed externally. Four 4‐0 polypropylene sutures with pledgets were placed-one over each commissure and one above the left main trunk-to secure this region. Once the bladder temperature reached 25°C, circulatory arrest was initiated, and retrograde cerebral perfusion was started via a cannula inserted in the superior vena cava. Using an open distal anastomosis technique, the distal aorta was trimmed just proximal to the brachiocephalic artery. A 15‐mm felt strip was placed externally and anchored with four 4‐0 polypropylene sutures with pledgets. A continuous 3‐0 polypropylene suture was then used to anastomose the vascular graft to the distal aorta. After systemic circulation was reestablished, a continuous 3‐0 polypropylene suture was employed to complete the proximal anastomosis between the graft and the ascending aorta.

The patient was successfully weaned off CPB without any complications, and the graft flow was satisfactory (mean graft flow, 51 mL/min; pulsatility index, 1.7; diastolic filling, 74%). The cross-clamp and CPB times were 80 min and 165 min, respectively.

The postoperative course was uneventful. The patient was extubated for 24 hours after surgery and discharged home on the 20th postoperative day. Postoperative TTE showed an improvement in EF of 45% (Video 2).

**Video 2:** Postoperative transthoracic echocardiography. Postoperative transthoracic echocardiography (TTE) showed an improvement in ejection fraction (EF) of 45%.

## Discussion

Acute AAD complicated by coronary malperfusion is a life-threatening condition requiring rapid and effective treatment. Neri et al. (2001) emphasized the critical importance of early coronary blood flow restoration and myocardial salvage in such cases [1]. Their research highlights the pivotal role of aggressive myocardial resuscitation and early surgical intervention in the effective management of these patients.

Imoto et al. (2013) also identified key mortality risk factors among patients with acute type A aortic and coronary artery dissections, highlighting the critical importance of early coronary revascularization [2]. Their study demonstrated that preoperative cardiopulmonary arrest and myocardial ischemia in the left coronary artery territory negatively affect survival outcomes, and that early revascularization through coronary stent placement was effective in preventing postoperative low cardiac output syndrome.

Under conventional repair-first management, coronary reperfusion is delayed, prolonging myocardial ischemia and potentially worsening outcomes, whereas a reperfusion-first strategy-PCI for dissection-related coronary malperfusion followed by central repair-was associated with a significant mortality reduction in the cohort reported by Uchida et al. (2018) [4].

The early restoration of coronary blood flow is critical in patients with coronary malperfusion secondary to aortic dissection. Francone et al. (2009) investigated the relationship between myocardial damage and time-to-reperfusion intervals and reported that a delay beyond 90 min in reperfusion following coronary occlusion significantly compromised myocardial preservation [7]. This principle also applies to acute AAD with coronary malperfusion, where prompt reperfusion is essential to minimize myocardial ischemia, reduce the risk of irreversible myocardial damage, and improve overall patient outcomes [8,9].

Recognizing the favorable outcomes reported in the PCI-first cases [10], we surmised that early coronary blood perfusion during surgery could also play a key role in improving surgical outcomes. In our patient, an immediate saphenous vein graft anastomosis to the LAD was performed, allowing blood flow from the CPB circuit to the LAD via a side branch, instead of following the conventional approach of cross-clamping the aorta and administering cardioplegia. After initiation of blood perfusion, there was marked improvement in the rigidity of the myocardium. The time from the start of surgery to blood reperfusion in the LAD was only 30 min, which contributed to minimal myocardial damage and significant postoperative improvement in cardiac function.

PCI for coronary malperfusion associated with aortic dissection requires advanced technical expertise, and prolonged PCI procedural times may lead to extended myocardial ischemia [11]. Therefore, surgical strategies must be tailored according to the expertise of the heart team. Our early blood perfusion surgical strategy has the advantage of achieving reperfusion, regardless of the coronary malperfusion mechanism. Consequently, it represents a viable and effective method for improving surgical outcomes in cases where surgery is prioritized in patients with acute AAD complicated by coronary ischemia.

In summary, this case highlights the importance of early coronary blood perfusion for the management of acute AAD with coronary malperfusion. Prioritizing relief from myocardial ischemia can significantly enhance surgical outcomes and patient survival. Our strategy of performing LAD bypass before aortic cross-clamping aligns with the favorable outcomes observed in PCI-first cases, offering a promising approach to improve the surgical results.

This report presents a single case, and additional data on the outcomes of this surgical approach are expected to be collected in the future.

## Conclusions

This case highlights the importance of early intervention in acute AAD with coronary malperfusion. Early blood perfusion of the LAD before aortic cross-clamping significantly improved patient prognosis. Prioritization of relief from myocardial ischemia leads to favorable outcomes. Timely coronary blood perfusion is crucial to prevent irreversible damage and improve outcomes. To obtain optimal results, optimal intervention timing and method must be determined on a case-by-case basis.
